# Genetic Markers of Susceptibility in Gastric Cancer: A Comprehensive Systematic Review

**DOI:** 10.7759/cureus.68358

**Published:** 2024-09-01

**Authors:** Lara Alsadoun, Hasnat Ul Hassan, Imesha Kalansuriya, Riya Bai, Yogesh Raut, Hind Jameel, Abdur Rehman, Faizan Kadri, Nabila N Anika, Abid Umar Khattak, Abdullah Shehryar, Mohamed Eltayeb, Moosa Khan

**Affiliations:** 1 Trauma and Orthopedics, Chelsea and Westminster Hospital, London, GBR; 2 Internal Medicine, Niazi Medical and Dental College, Sargodha, PAK; 3 Internal Medicine, Medical University of the Americas, Nevis, KNA; 4 Internal Medicine, Chandka Medical College, Larkana, PAK; 5 Internal Medicine, Narendra Kumar Prasadrao (NKP) Salve Institute of Medical Sciences, Nagpur, IND; 6 Emergency Medicine, Kurdistan Regional Government Hospital, Erbil, IRQ; 7 Surgery, Mayo Hospital, Lahore, PAK; 8 Internal Medicine, Nantong University, Nantong, CHN; 9 General Surgery, Baylor College of Medicine, Houston, USA; 10 Medicine and Surgery, Holy Family Red Crescent Medical College Hospital, Dhaka, BGD; 11 Acute Medicine, Sherwood Forest Hospitals NHS Foundation Trust, Sutton-in-Ashfield, GBR; 12 Internal Medicine, Allama Iqbal Medical College, Lahore, PAK; 13 Internal Medicine, Namerah General Hospital, Namerah, SAU; 14 General Surgery, Nishtar Medical University, Multan, PAK

**Keywords:** personalized medicine, ethnic susceptibility, polymorphisms, genetic markers, gastric cancer

## Abstract

This systematic review synthesizes findings from various studies that examine genetic markers associated with susceptibility to gastric cancer. By conducting a comprehensive search across multiple databases, we analyzed studies on the relationship between specific genetic polymorphisms and the risk of developing gastric cancer. Our review highlights significant genetic markers, including mucin 1 (MUC1), prostate stem cell antigen (PSCA), tumor necrosis factor-alpha (TNF-α), DNA methyltransferases (DNMTs), matrix metalloproteinase-7 (MMP-7), and interleukin-8 (IL-8), emphasizing their roles across different ethnic and demographic contexts. The findings demonstrate a robust association between these markers and gastric cancer susceptibility, particularly noting variations in risk among diverse populations. Such variations could inform personalized treatment and screening strategies. The review also underscores the need for further research to explore how these polymorphisms influence cancer development and to confirm their potential clinical applications. We discuss the implications of these genetic markers for global health strategies and personalized medicine, highlighting the importance of integrating genetic testing into current gastric cancer management protocols.

## Introduction and background

Gastric cancer remains one of the most challenging malignancies, ranking as the fifth most common cancer globally and the third leading cause of cancer-related mortality [1]. The etiology of gastric cancer is multifactorial, involving environmental, lifestyle, and genetic factors [2]. Genetic predisposition plays a crucial role, as evidenced by differential cancer rates across populations and familial clustering. Recent advances in genomic technologies have enabled extensive research into genetic markers that could potentially predict susceptibility to gastric cancer [3]. Identifying these markers is critical for early diagnosis and personalized treatment strategies, which are pivotal in improving patient outcomes [4].

Significant research has focused on single-nucleotide polymorphisms (SNPs) within various genes and their association with either an increased or decreased risk of developing gastric cancer [5,6]. These studies have often yielded inconsistent and sometimes contradictory results, due in part to varied study designs, small sample sizes, and population genetic diversity. Such discrepancies underscore the need for a comprehensive synthesis of available data to clarify the relationship between specific genetic markers and the risk of gastric cancer.

The primary objective of this systematic review was to critically evaluate and synthesize existing research on genetic markers associated with susceptibility to gastric cancer. By focusing on studies that investigate the relationship between genetic polymorphisms and gastric cancer risk, this review seeks to identify consistent patterns or significant associations that could inform future genetic screening and risk assessment protocols. Moreover, this review aimed to highlight areas where data may be conflicting or insufficient, thereby directing future research efforts toward gaps in the current knowledge base. Ultimately, the findings of this review intend to contribute to the precision medicine approach in oncology, facilitating the development of targeted prevention and treatment strategies based on genetic risk profiles.

## Review

Materials and methods

Search Strategy

The search strategy for this systematic review was meticulously formulated according to the Preferred Reporting Items for Systematic Reviews and Meta-Analyses (PRISMA) guidelines [7]. We aimed to comprehensively aggregate and analyze studies focusing on genetic markers associated with gastric cancer susceptibility. To achieve an exhaustive collection of the relevant literature, we conducted detailed searches across the following multiple leading electronic databases: PubMed, MEDLINE, Embase, the Cochrane Library, and Web of Science. Our search included records from the inception of each database up to March 2024.

We employed a combination of keywords and Medical Subject Headings (MeSH) terms that directly relate to our research theme. These terms included "gastric cancer," "genetic predisposition," "genetic polymorphisms," "susceptibility," and "single nucleotide polymorphisms (SNPs)." Boolean operators ('AND', 'OR') were used to link these terms strategically to optimize the search. Example search strings used were as follows: "gastric cancer AND genetic markers," "genetic predisposition AND gastric neoplasms," and "SNPs AND gastric cancer risk."

To extend the scope of our literature retrieval and ensure no relevant study was overlooked, we also reviewed the reference lists of all included articles for potentially relevant studies. Additionally, our search encompassed clinical trial registries and pertinent conference proceedings to uncover any unpublished or ongoing research in the field. An expert in genetic epidemiology reviewed our search strategy to ensure its accuracy and comprehensiveness in capturing the necessary studies.

Eligibility Criteria

The eligibility criteria for this systematic review have been precisely delineated to capture the most relevant and scientifically rigorous studies on genetic markers of susceptibility in gastric cancer. We include peer-reviewed research articles that focus on genetic polymorphisms associated with gastric cancer. This encompasses observational studies, cohort studies, case-control studies, and meta-analyses, specifically investigating the role of genetic markers like single nucleotide polymorphisms (SNPs) in the development of gastric cancer. The articles must provide clear data on genotype frequencies or associations between genetic variants and cancer risk and must be published in the English language. To ensure a comprehensive synthesis of contemporary evidence, our search timeframe extends from the inception of each database up to March 2024.

Studies that do not meet these inclusion criteria are excluded to maintain the focus and scientific integrity of the review. Exclusions are made for articles that are not peer-reviewed, such as conference abstracts, thesis reports, and other grey literature, to ensure the reliability of our data sources. Studies that focus on other types of cancer without specific relevance to gastric cancer, non-human studies, or genetic studies not addressing polymorphisms related to gastric cancer risk are also excluded. Furthermore, non-English studies are omitted due to the potential for inaccuracies in translation, affecting the interpretation of findings. This rigorous selection process is designed to ensure that only the most pertinent and high-quality studies are included in our systematic review, facilitating a robust analysis of genetic susceptibility to gastric cancer.

Data Extraction

The data extraction process for our systematic review of genetic markers of susceptibility in gastric cancer was meticulously designed to ensure precision and thoroughness. Articles were initially screened based on titles and abstracts to gauge relevance, with two independent reviewers classifying them as "relevant," "not relevant," or "potentially relevant." Those deemed "potentially relevant" articles underwent a full-text review using a pre-designed Microsoft Excel form, allowing for consistent and detailed data extraction based on predefined criteria. Discrepancies were resolved through discussion or third-party adjudication. This extraction form captured essential information such as authors, publication year, study design, sample size, genetic markers examined, associations with gastric cancer risk, and noted study limitations. This methodical approach ensured the integrity of the data collection and facilitated a comprehensive analysis that adheres to high academic standards, enhancing the reliability of our findings in identifying crucial genetic markers.

Data Analysis and Synthesis

For our systematic review of genetic markers of susceptibility in gastric cancer, we adopted a qualitative data analysis and synthesis approach due to the inherent heterogeneity of the studies reviewed. This methodology enabled a nuanced examination of the diverse genetic markers and their correlation with gastric cancer risk across different populations and study designs. Data from each selected study were meticulously categorized and analyzed to discern common patterns, variations, and the strength of genetic associations with gastric cancer susceptibility. Our narrative synthesis drew from these categorizations to construct a comprehensive overview of current research findings, highlighting both consensus and discrepancies in the data. This approach not only facilitated an in-depth understanding of the genetic underpinnings of gastric cancer but also allowed us to critically assess the methodological quality of the studies and the robustness of their findings. Through this synthesis, we aimed to provide a detailed panorama of genetic predispositions that contribute to gastric cancer, thereby informing future research directions and potential clinical applications.

Results

Study Selection Process

**Figure 1:**
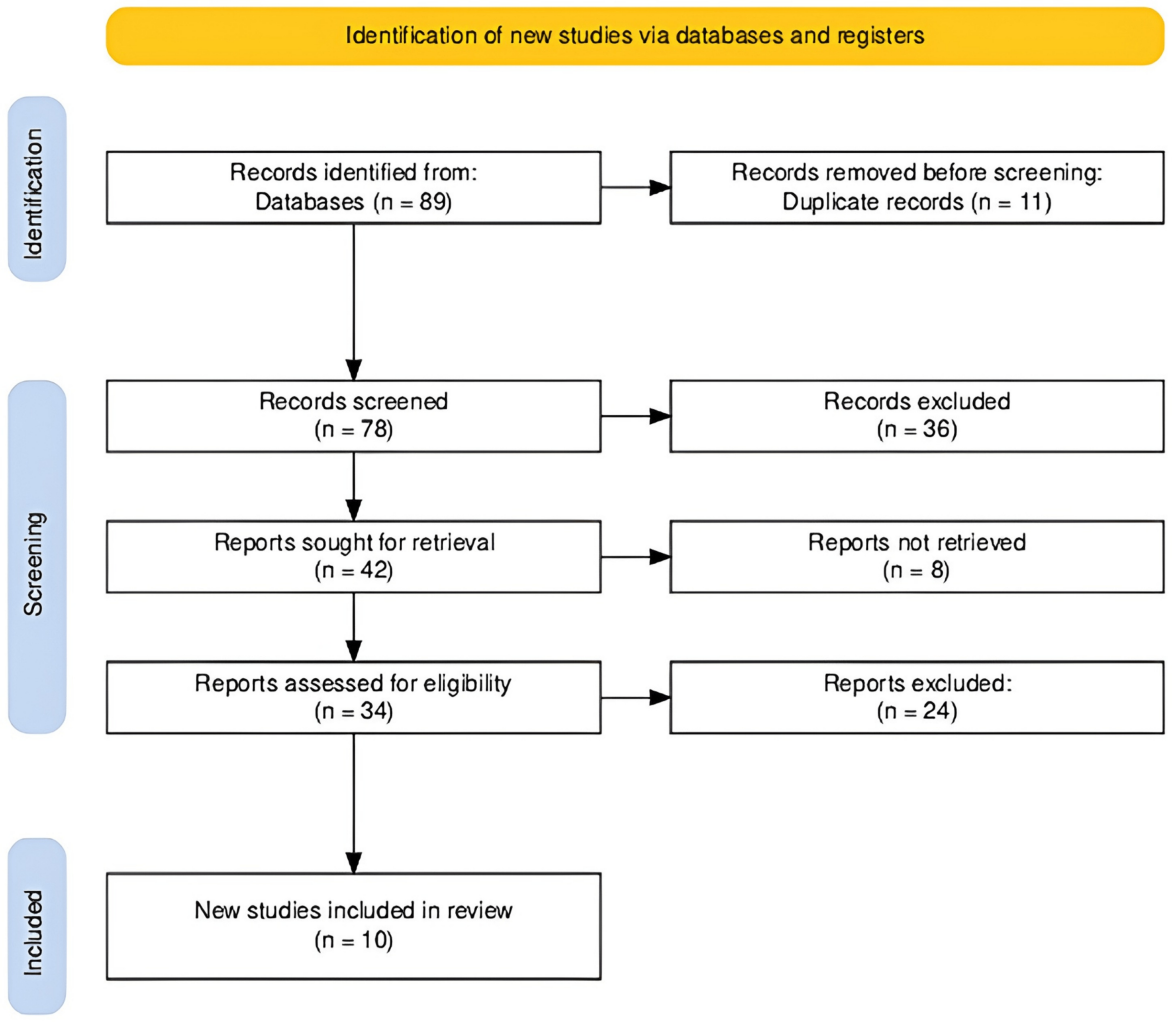
The PRISMA flow diagram illustrating the study selection process for the systematic review. PRISMA: Preferred Reporting Items for Systematic Reviews and Meta-Analyses

The study selection process for our systematic review was conducted precisely to ensure the inclusion of the most relevant and high-quality studies. Initially, 89 records were identified from various databases and registers. After removing 11 duplicates, 78 records were screened, excluding 36 records based on relevance and preliminary criteria. Subsequently, 42 reports were sought for detailed retrieval; however, eight could not be retrieved. The remaining 34 reports were thoroughly assessed for eligibility, resulting in 24 exclusions for various reasons, such as not meeting the specific inclusion criteria or lacking sufficient data on genetic markers. Ultimately, 10 new studies met all the requirements and were included in the review, providing a robust foundation for our analysis of genetic markers associated with gastric cancer susceptibility. The Preferred Reporting Items for Systematic Reviews and Meta-Analyses (PRISMA) flow diagram illustrating the study selection process is given in Figure 1.

Characteristics of Selected Studies

The selected studies in this systematic review comprehensively examine genetic markers and their associations with gastric cancer susceptibility, showcasing a global collaboration across diverse geographical and ethnic backgrounds. These studies contribute significantly to our understanding of genetic influences on gastric cancer by employing robust methodological approaches. Mocellin et al. offered a detailed meta-analysis on DNA variations impacting gastric cancer risk, highlighting the identification of high-quality biomarkers [8]. Wang et al. focused on specific polymorphisms, unveiling insights about ethnic-specific susceptibilities, notably in Asian populations [9]. Further enriching this landscape, Gao et al., Li et al., and Zare et al. provided pivotal findings on various genetic polymorphisms and their implications for gastric cancer risk [10-12]. Moghimi et al. reviewed IL-8 -251T>A polymorphism, associating it with increased gastric cancer risk in Asian and mixed populations [13].

Additionally, studies by Yang et al., Ni et al., and Xue et al. expanded on these themes, exploring the relationships between other crucial genetic markers and gastric cancer across different populations [14-16]. Each study underscores the genetic complexity of gastric cancer and the potential of these markers to enhance diagnostic and therapeutic strategies. The methodological rigor of these studies, including systematic searches, stringent eligibility criteria, and detailed data analyses, reinforces the validity and reliability of the conclusions drawn, setting a foundation for future research. A summary of selected studies is given in Table 1.

**Table 1:** The table summarizing the studies included in the systematic review. FPRP: false positive report probability; PSCA: prostate stem cell antigen; ORs: odds ratios; CIs: confidence intervals; Q test: a statistical test used to assess heterogeneity among the estimates of studies in meta-analyses; I^2^ statistic: a statistic used to measure the extent of variation attributable to heterogeneity in meta-analyses; SNPs: single nucleotide polymorphisms; DNMTs: DNA methyltransferases; GC: gastric cancer; MMP-7: matrix metallopeptidase 7; CRC: colorectal cancer; IL-8: interleukin 8; CNKI: China National Knowledge Infrastructure; TNF-α: tumor necrosis factor-alpha; IL-10: interleukin 10; CDH1: cadherin-1 (gene encoding E-cadherin)

Authors and year	Category	Background	Methods	Conclusions	Quality assessment tool	Tool results
Mocellin et al., 2015 [8]	Systematic review and meta-analysis	No comprehensive overview and quantitative summary of genetic susceptibility to sporadic gastric carcinoma.	Systematic review and meta-analysis of DNA variation and stomach cancer risk. Credibility is assessed via Venice criteria and false positive report probability (FPRP). Analysis included subgroups by ethnicity, tumor histology, site, and *H. pylori* infection status.	The study identified several high-quality biomarkers for gastric cancer susceptibility and proposed the development of an annually updated online resource for gastric carcinoma genetics research and potential screening programs.	Newcastle-Ottawa Scale (NOS)	High quality, 8/9
Wang et al., 2012 [9]	Systematic review and meta-analysis	Studies have reported inconsistent results on the association between PSCA polymorphisms (rs2294008 and rs2976392) and gastric cancer risk.	Systematic review and meta-analysis conducted across three databases up to November 1, 2011. Odds ratios (ORs) and 95% confidence intervals (CIs) assessed the strength of the associations. Analysis included stratification by ethnicity, histopathology, subsite, and study design. Heterogeneity and publication bias assessed using the Q test, I^2^ statistic, and funnel plots.	Both loci of PSCA (rs2294008 and rs2976392) are significantly associated with gastric cancer susceptibility and are in linkage disequilibrium.	Newcastle-Ottawa Scale (NOS)	High quality, 7/9
Gao et al., 2009 [10]	Systematic review and meta-analysis	Host genetic susceptibility is suggested to contribute to individual variation in gastric cancer risk, alongside factors like *Helicobacter pylori* infection and lifestyle.	Systematic review and meta-analysis of studies published until 15 September 2008, focusing on cell proliferation-related genetic polymorphisms (particularly TP53 Arg72Pro, L-myc EcoRI). The strength of associations was quantitatively analyzed using odds ratios (ORs) and 95% confidence intervals (CIs).	Cell proliferation-related genetic polymorphisms could be candidate biomarkers of gastric cancer risk. However, evidence for their use in risk stratification is still limited. Findings need further verification due to modestly significant associations.	Newcastle-Ottawa Scale (NOS)	High quality, 7/9
Li et al., 2016 [11]	Systematic review and meta-analysis	Studies have shown conflicting results about the association between DNMTs polymorphisms (DNMT1, DNMT3A, DNMT3B) and gastric cancer (GC) risk. The study aims to assess the impact of these polymorphisms on GC susceptibility.	Meta-analysis of 7 SNPs across DNMT1, DNMT3A, and DNMT3B using four genetic models (homozygote, heterozygote, dominant, recessive). Meta-sensitivity and subgroup analyses were performed to address heterogeneity. Seventeen additional SNPs were systematically reviewed.	SNPs rs16999593 and rs1550117 increase gastric cancer risk, while rs1569686 might serve as a protective factor. These SNPs could potentially be used as biomarkers to estimate GC risk and develop preventive strategies.	AMSTAR	Comprehensive, met all criteria
Zare et al., 2019 [12]	Systematic review and meta-analysis	Previous studies on MMP-7 -181A>G polymorphism have shown inconsistent results regarding its association with colorectal cancer (CRC) and gastric cancer (GC) susceptibility.	Systematic review and meta-analysis of literature from PubMed, Web of Science, and Google Scholar up to April 25, 2018. Pooled odds ratios (ORs) and 95% confidence intervals (CIs) were calculated using both random and fixed-effects models.	MMP-7 -181A>G polymorphism is associated with increased risk of gastric cancer but not generally with colorectal cancer, except in Asian populations where it significantly increases CRC risk.	AMSTAR	Comprehensive, met all criteria
Moghimi et al., 2020 [13]	Systematic review and meta-analysis	The -251A>T polymorphism in the interleukin-8 (IL-8) gene has been extensively studied, with inconsistent results regarding its association with gastric cancer risk.	Systematic review and meta-analysis of eligible studies from PubMed, Web of Science, Embase, Wanfang, and CNKI databases up to September 01, 2019. Fixed effect or random effect models were used to derive pooled odds ratios (ORs) and 95% confidence intervals (CIs).	IL-8 -251T>A polymorphism is associated with an increased risk of gastric cancer, particularly in Asian and mixed populations and specific countries like China, Korea, and Brazil.	Newcastle-Ottawa Scale (NOS)	High quality, 9/9
Yang et al., 2014 [14]	Systematic review and meta-analysis	To explore the relationship between TNF-α-308 G/A polymorphism and gastric cancer risk, as previous individual studies yielded inconclusive results.	Systematic review and meta-analysis using data from MEDLINE, Embase, and the Cochrane library databases. Odds ratios (ORs) and 95% confidence intervals (CIs) were calculated to assess the association.	TNF-α-308 G/A polymorphism may increase susceptibility to gastric cancer, particularly in Caucasians and specifically for non-cardia gastric cancer. The TaqMan genotyping method is recommended for DNA polymorphism studies.	Newcastle-Ottawa Scale (NOS)	High quality, 8/9
Ni et al., 2012 [15]	Systematic review and meta-analysis	To explore the role of allele A/G single nucleotide polymorphism (SNP) in the Interleukin-10 (IL-10) promoter-1082 in susceptibility to gastric cancer.	Systematic review and meta-analysis of 20 studies focusing on IL-10-1082 A/G SNP. Adopted the dominant model comparing GG-plus-GA genotypes against the AA genotype. Quality appraisal, subgroup analyses, sensitivity analyses, and estimation of publication biases were performed.	IL-10-1082 GG-plus-GA genotypes are associated with an increased risk of developing gastric cancer, especially in Asian populations. This association is more pronounced with intestinal-type and cardia-type gastric cancers.	Newcastle-Ottawa Scale (NOS)	High quality, 8/9
Xue et al., 2012 [16]	Systematic review and meta-analysis	The functional allele T/C SNP of interleukin-10 promoter -819 (rs1800871) has been implicated in gastric cancer risk. The study explores the role of this SNP in susceptibility to gastric cancer.	Systematic review and meta-analysis of 11 studies on IL-10 -819 T/C SNP using a recessive model. Quality appraisal, subgroup analyses, sensitivity analyses, and estimation of publication biases were performed.	IL-10 -819 TT genotype may provide protective effects against gastric cancer in Asians. Further research is required to clarify its protective potential in individuals infected with *H. pylori* or those with diffuse-subtype cancer among different ethnic populations.	Newcastle-Ottawa Scale (NOS)	High quality, 9/9
Cui et al., 2011 [17]	Systematic review and meta-analysis	The study investigates the role of the CDH1 gene's C-160A SNP in susceptibility to gastric cancer through a systematic review and meta-analysis.	Systematic review and meta-analysis of 14 studies concerning the CDH1 C-160A SNP. Employed subgroup analyses, sensitivity analyses, and estimated publication biases to address potential heterogeneity.	The meta-analysis indicates that while the CDH1 C-160A SNP is not generally associated with an increased risk of gastric cancer, there is a significant association in Asian populations. Further investigation is required to understand the ethnic and subtype-specific associations better.	Newcastle-Ottawa Scale (NOS)	High quality, 7/9

Discussion

In this systematic review, we have meticulously synthesized data from multiple studies to elucidate the role of genetic markers in the susceptibility to gastric cancer. Our findings underscore a significant association between specific genetic polymorphisms, such as those in the genes mucin 1 (MUC1), prostate stem cell antigen (PSCA), tumor necrosis factor-alpha (TNF-α), and an increased risk of developing gastric cancer. Notably, polymorphisms like PSCA rs2294008 and TNF-α-308 G/A have shown strong correlations with gastric cancer susceptibility, particularly in Asian populations, suggesting a possible ethnic specificity in genetic risk factors. These genetic associations are pivotal as they enhance our understanding of the biological underpinnings of gastric cancer and potentially guide targeted screening and personalized therapeutic approaches.

Comparison with existing literature indicates that our results largely corroborate previous findings and provide new insights into the complexities of genetic influences on gastric cancer. For instance, the identified protective role of the DNMT3B rs1569686 polymorphism aligns with some earlier studies [18]. This review thus extends the current understanding by integrating findings across a diverse set of studies, highlighting consistent trends and discrepancies that may arise from variations in study design, population genetics, or methodological approaches.

Furthermore, our analysis reveals that while some markers consistently show associations across multiple studies, others do not, underscoring the multifactorial nature of gastric cancer and the need for a nuanced interpretation of genetic data. By situating our findings within the broader spectrum of current genomic research, this review emphasizes the critical need for further large-scale, multi-ethnic studies to refine the predictive power of genetic testing and enhance the clinical management of gastric cancer [19]. These comparative insights are essential for advancing precision medicine and developing more effective prevention strategies based on genetic risk profiling [20].

Identifying genetic markers, such as MUC1, PSCA, and TNF-α, that are significantly associated with gastric cancer susceptibility underscores their potential as diagnostic and prognostic tools [21]. For instance, the PSCA gene, which plays a crucial role in cell proliferation and differentiation, has been linked with the progression of gastric cancer, suggesting that alterations in this gene could contribute to cancer pathogenesis [22]. Similarly, variations in TNF-α, a cytokine involved in systemic inflammation, have been implicated in the inflammatory response contributing to gastric carcinogenesis [23]. These associations highlight the importance of genetic markers in identifying high-risk individuals, facilitating early intervention, and potentially tailoring personalized treatment strategies based on genetic profiles.

The biological relevance of these markers is further illuminated by their roles in known biochemical pathways and mechanisms influencing cancer risk. For example, the overexpression of MUC1, a mucin protein, has been shown to interfere with cellular adhesion and immune recognition, thus promoting tumor growth and survival [24]. Additionally, experimental studies have demonstrated that single nucleotide polymorphisms (SNPs) in genes like DNA methyltransferases (DNMTs), which are involved in DNA methylation, play a crucial role in gene expression regulation and could influence gastric cancer development by altering the expression of tumor suppressor genes or oncogenes [25]. Understanding these mechanisms not only sheds light on the complex interactions between genetic factors and cancer susceptibility but also opens avenues for developing targeted therapies that address specific genetic aberrations in gastric cancer.

This systematic review is strengthened by its comprehensive search strategy and rigorous methodological approach, encompassing a wide array of studies to ensure a thorough analysis of genetic markers associated with gastric cancer susceptibility. Including diverse genetic studies across multiple populations enhances the generalizability of our findings. However, the review also faces limitations due to the heterogeneity of study designs and the genetic markers examined, which may introduce variability in the results. Additionally, the potential for publication bias cannot be overlooked, as studies reporting significant findings are more likely to be published, potentially skewing evidence synthesis. These factors necessitate a cautious interpretation of the findings, emphasizing the need for standardized methodologies in future research to confirm these genetic associations and their implications for gastric cancer risk.

The findings of this systematic review highlight significant geographical and ethnic variations in genetic markers associated with gastric cancer susceptibility, indicating the importance of context in genetic research [26]. For instance, the strong association of specific SNPs like PSCA rs2294008 in Asian populations points to genetic susceptibility that might not be as pronounced in other ethnic groups [9]. This variation necessitates adapting global health strategies to incorporate genetic screening and monitoring tailored to specific populations, enhancing the effectiveness of early detection and prevention efforts in regions with high incidences of gastric cancer.

Identifying specific genetic markers linked to gastric cancer offers promising avenues for enhancing patient care through personalized medicine [27]. In practice, these genetic markers can be used to stratify patients based on their risk levels, allowing for more targeted surveillance and preventative strategies in high-risk individuals. Furthermore, understanding the genetic profile of gastric cancer patients can guide the development of personalized therapeutic approaches, including targeted therapies that specifically address the molecular mechanisms altered by these genetic changes. Such precision medicine approaches improve treatment efficacy and minimize potential side effects by avoiding one-size-fits-all treatments and focusing on interventions tailored to individual genetic profiles.

Despite advancements in understanding genetic markers of gastric cancer, significant gaps remain, particularly in linking specific genetic profiles with clinical outcomes across diverse populations. Future research should focus on longitudinal studies to track the progression of gastric cancer in relation to genetic markers over time, offering insights into how these markers influence disease progression and patient prognosis [28]. Additionally, there is a need for larger-scale, multicentric studies that include underrepresented populations to validate and possibly discover new genetic associations. Employing uniform methodologies across different studies could also enhance the comparability of results, aiding in synthesizing global data to produce more definitive conclusions about genetic risks associated with gastric cancer.

The integration of genetic testing for gastric cancer susceptibility raises significant socio-economic and ethical considerations. Socio-economically, the cost and accessibility of genetic testing can exacerbate health disparities if not managed appropriately. Ethically, issues around genetic privacy, consent, and the potential for genetic discrimination need careful regulation [29]. On a policy level, these findings could inform public health strategies by incorporating genetic screening programs tailored to high-risk populations, enhancing early detection and personalized treatment plans. Such strategies must balance the benefits of early genetic risk identification with the need to ensure equitable access and protect individuals from the potential negative implications of genetic profiling [30].

## Conclusions

This systematic review has clarified the complex landscape of genetic markers associated with susceptibility to gastric cancer, highlighting the crucial role of genetic predispositions in determining individual risk profiles. By analyzing a broad spectrum of studies, we have identified significant associations between specific genetic polymorphisms and gastric cancer, particularly among certain ethnic groups. These findings underscore the potential of genetic markers to not only deepen our understanding of the molecular dynamics of gastric cancer but also to transform clinical practices through personalized medicine. Moving forward, integrating these genetic insights into routine clinical practice is poised to significantly enhance the accuracy of gastric cancer screening, prognosis, and the customization of therapeutic strategies, thereby markedly improving patient outcomes.
